# Glioma Stem Cells and Immunotherapy for the Treatment of Malignant Gliomas

**DOI:** 10.1155/2013/673793

**Published:** 2013-05-15

**Authors:** Masahiro Toda

**Affiliations:** Department of Neurosurgery, Keio University School of Medicine, 35 Shinanomachi, Shinjuku-ku, Tokyo 160-8582, Japan

## Abstract

Stem cell research has led to the discovery of glioma stem cells (GSCs), and because these cells are resistant to chemotherapy and radiotherapy, analysis of their properties has been rapidly pursued for targeted treatment of malignant glioma. Recent studies have also revealed complex crosstalk between GSCs and their specialized environment (niche). Therefore, targeting not only GSCs but also their niche may be a principle for novel therapies of malignant glioma. One possible novel strategy for targeting GSCs and their niches is immunotherapy with different antitumor mechanism(s) from those of conventional therapy. Recent clinical studies of immunotherapy using peptide vaccines and antibodies have shown promising results. This review describes the recent findings related to GSCs and their niches, as well as immunotherapies for glioma, followed by discussion of immunotherapies that target GSCs for the treatment of malignant glioma.

## 1. Introduction

Gliomas are the most common histological type of malignant brain tumor and share the feature of having some degree of glial differentiation. Over 80% of gliomas are astrocytic tumors, including glioblastoma (GBM), the most malignant glioma [1]. Gliomas are characterized by their infiltrating nature, and extensive invasion into the surrounding normal brain tissue is often observed. Despite advances in treatment strategies, the prognosis for GBM patients remains very poor, and the resistance of GBM against treatment causes a high rate of tumor recurrence.

The cancer stem cell (CSC) hypothesis provides an explanation for the therapeutic resistance and ability to regenerate tumors from a small population of cells. According to this hypothesis, only CSCs exhibiting stem-like characteristics can propagate and reinitiate the tumor. Recent studies support the existence of CSCs in GBM [2, 3], and a small number of glioma stem cells (GSCs) with resistances against conventional chemotherapy and radiotherapy are sufficient to give rise to recurrent tumors [4]. In addition, because of their ability for multipotent differentiation and tumor initiation, GSCs can generate heterogeneous tumor masses as GBM. Since the discovery of GSCs, research for the treatment of GBM has focused on the identification of intrinsic molecular pathways involved in regulation of their stemness and tumorigenicity. However, it has become clear that GSCs are tightly regulated by specialized microenvironments (niches) within tumors, namely, vascular and hypoxic niches [5]. Furthermore, GSCs do not simply receive signals from the surrounding niche but are also capable of modulating their niches through complex crosstalk [6]. GSCs play a key role in shaping vascular niches through hypoxia-dependent stimulation of new blood vessel formation (angiogenesis), recruitment of endothelial progenitor cells, and direct trans-differentiation into endothelial cells. Furthermore, GSCs and the vascular niche represent integral parts of the tumor, which facilitate invasion and expansion. Therefore, understanding the interactions between GSCs and their niche is important for new therapeutic approaches.

Recently, various immunotherapies have been attempted and some clinical studies have shown promising efficacy for the treatment of GBM [7–12]. In particular, cancer vaccines with epitope peptides for induction of cytotoxic T lymphocyte (CTL) responses in patients have shown encouraging results. Because GSCs are resistant to chemotherapy, more investigators are turning to immunotherapeutic strategies that target GSCs. Recent preclinical studies have also shown the effectiveness of immunotherapies targeting GSCs [13–16]. To design a rational immunotherapy against GBM, clear knowledge of GSCs and their niches is required. This review describes recent findings related to GSCs and their niches, as well as immunotherapies for treating glioma, followed by discussion of new immunotherapeutic strategies that target GSCs and their niche. 

## 2. Glioma

Gliomas are the most frequent primary tumor that arises in the brain, and the World Health Organization (WHO) classifies gliomas according to different grades of malignancy (I–IV) [17]. Malignant gliomas are generally high grade (III and IV) in the WHO classification and the most malignant form of glioma is GBM. GBMs are heterogeneous tumors in both appearance and gene expression and exhibit the greatest range of genetic abnormalities. Recent genomic studies have revealed a set of core signaling pathways commonly activated in GBM, namely, p53, retinoblastoma, and receptor tyrosine kinase pathways [18, 19]. Moreover, The Cancer Genome Atlas project has provided somatic mutation information that revealed potential new roles for known tumor suppressors/oncogenes in GBM as well as new cancer driver genes.

According to differences in clinical courses and their gene expression profiles, GBMs are subclassified into primary and secondary GBMs [20, 21], although the histology of both types of GBM is identical. Primary GBM occurs *de novo* and is characterized by epidermal growth factor receptor (EGFR) amplification/overexpression and PTEN mutation [22, 23]. Secondary GBM is a result of malignant progression of lower grade tumors and mutation of the TP53 tumor suppressor gene, which appears to be an early event during the development of GBM. Recently, somatic mutations in the isocitrate dehydrogenase 1 (IDH1) or IDH2 gene have been identified in the majority of WHO grade II and III gliomas and secondary GBMs [18, 24]. Although the mechanism through which IDH1/2 mutations transform cells is far from clear, gliomas with IDH1 mutations show a significantly higher frequency of the CpG island methylator phenotype as well as increased histone demethylation [25]. 

Alkylating agents, such as nimustine, carmustine, lomustine, procarbazine, and temozolomide (TMZ), which are commonly used for the treatment of GBMs, cause DNA damage and induce apoptosis in tumor cells. O6-methylguanine-methyltransferase (MGMT) is a DNA repair gene and high levels of MGMT activity in tumors are associated with resistance against alkylating agents such as TMZ [26]. The extent of methylation of the promoter region of the MGMT gene correlates with the outcome of treatment with alkylating agents [27]. Thus, molecular information is becoming more useful for making a clinical diagnosis and formulating treatment plans. 

## 3. Angiogenesis in Glioma

Angiogenesis is a fundamental event in the process of glioma growth and invasion. The current model of tumor angiogenesis suggests that this process involves recruitment of sprouting vessels from existing blood vessels and incorporation of endothelial progenitors into the growing vascular bed [28]. Tumor angiogenesis involves proliferation, migration, and invasion of endothelial cells, organization of endothelial cells into functional tubular structures, and maturation of vessels. The angiogenic process is a highly complex, dynamic process regulated by various pro- and antiangiogenic molecules. One of the major pathways in angiogenesis involves the vascular endothelial growth factor (VEGF) family and their receptors.

Hypoxia has long been known as a major stimulator of angiogenesis in GBM [29]. Necrotic foci surrounded by pseudopalisading cells are a configuration relatively unique to GBM. Pseudopalisading cells are a series of actively migrating glioma cells around severely hypoxic regions, which overexpress hypoxia-inducible factors (HIFs) and secrete VEGF [30]. VEGF is upregulated by hypoxia and stimulates not only angiogenesis, but also cell migration towards viable vessels. Recent evidence suggests that hypoxia and HIFs play a critical role in maintenance of the GSC fraction in GBM by creating a microenvironment that provides the essential cellular interactions and environmental signals needed to retain the stemness of GSCs [31]. 

### 3.1. VEGF Family Members in Glioma

The VEGF family consists of five members: VEGF-A (hereafter called VEGF), VEGF-B, VEGF-C, VEGF-D, and placental growth factor (PIGF). VEGF family ligands have different affinities for the three VEGF tyrosine kinase receptors: VEGF receptor (VEGFR-) 1, VEGFR-2, and VEGFR-3.

VEGF binds to VEGFR-1 and VEGFR-2, which induces vascular permeability and functions as an endothelial cell mitogen and survival factor, as well as an inducer of endothelial cell and monocyte migration [32]. Moreover, VEGF is a strong angiogenic effector under most physiological and pathological conditions including glioma [33]. The roles of VEGF-B and PIGF, which bind to VEGFR-1, may be redundant. However, recent studies revealed that VEGF-B is a potent survival factor for blood vessels, and inhibition of VEGF-B leads to pathological angiogenesis by abolishing blood vessel survival in animal models [34]. In addition, loss of PlGF impairs angiogenesis during ischemia, inflammation, wound healing, and tumor growth [35]. Therefore, VEGF-B and PlGF may play important roles in pathological states.

VEGF-C binds to VEGFR-2 and VEGFR-3 and is involved in developmental lymphangiogenesis and the maintenance of adult lymphatic vasculature [33]. Although VEGF-C is not normally expressed in the brain, recent reports indicate high expression of VEGF-C in malignant glioma, suggesting a role in glioma angiogenesis [36]. VEGF-D binds to both VEGFR-2 and VEGFR-3 and is involved in developmental lymphangiogenesis and adult lymphatic vasculature [33]. VEGF-C and VEGF-D bind to VEGFR-2, and they might also play a role in angiogenesis, particularly during pathological states such as tumor growth. However, the role of these ligands in tumor angiogenesis is unclear. Similar to VEGF-C, VEGF-D is also highly expressed in GBM, but not in the normal brain, suggesting that these ligands contribute to glioma angiogenesis [36].

### 3.2. VEGF Receptors in Glioma

There are three types of receptor tyrosine kinases that mediate the angiogenic functions of VEGF family members [32]. VEGFR-1 and VEGFR-2 were originally identified on endothelial cells, and VEGFR-3 was identified on lymphatic endothelial cells.

Initially, VEGFR-1 was considered to be a negative regulator of VEGF activity by acting as a decoy receptor for VEGF. VEGFR-1 is also expressed by monocytes, macrophages, and other bone-marrow-derived progenitor cells (myeloid cells), which mediates the migration of bone-marrow-derived cells into cancer tissues as well as the recruitment of endothelial progenitor cells, resulting in tumor growth and angiogenesis [37]. In addition, VEGFR-1 is expressed by subsets of CSCs, which results in tumor cell survival and growth [32]. During pathological conditions such as tumorigenesis, recent studies have shown that VEGFR-1 is a potent, positive regulator of angiogenesis [35]. Therefore, the current evidence suggests that the function of VEGFR-1 differs in various states of physiological and pathological conditions, and in the cell types expressing VEGFR-1.

VEGFR-2 is the major mediator of VEGF-induced angiogenic signaling. The functions of VEGFR-2 include endothelial cell survival, migration, proliferation, and vascular permeability. This receptor has the most important role in vessel formation during both physiological and pathological conditions [35]. In addition to endothelial cells, recent studies have shown that VEGFR-1 and VEGFR-2 are also expressed in glioma cells [38]. A functional VEGF/VEGFR-2 autocrine loop has been found in subsets of leukemia for tumor survival and migration [39]. These observations suggest that VEGF may also have another role in cancer through the stimulation of VEGFRs on tumor cells.

VEGFR-3 binds to both VEGF-C and VEGF-D and is important for the regulation of normal and tumor lymphangiogenesis [35]. During development and postnatally, VEGFR-3 expression is limited to lymphatic endothelial cells. VEGFR-3 is not expressed in the brain, but recent studies have shown that VEGFR-3 is highly expressed together with VEGF-C and VEGF-D in malignant glioma, suggesting that these ligands and VEGFR-3 contribute to glioma angiogenesis [36, 40].

## 4. Glioma Stem Cells

CSCs have been defined by the American Association for Cancer Research workshop on CSCs as a subpopulation of cells in the tumor, which have a self-renewal capacity and can give rise to heterogeneous cancer cells that comprise the tumor [41]. The presence of CSCs in human GBM was first reported in 2003 [3]. Similar to neural stem cells (NSCs), GSC characteristics include the ability to generate clonally derived cells and self-renew, as well as the potential to differentiate into multiple lineages (neural, astrocytic, and oligodendroglial) [42]. GSCs express several important determinants of NSC fate, which are used for their identification, including CD133, Nestin, Sox2, CD15, and Musashi [43]. 

Similar to NSC culture, GSCs were initially cultured as spheroids in serum-free medium containing epidermal growth factor and fibroblastic growth factor [4, 43]. Tumor cells grown under an NSC culture condition can be stably maintained and form tumors similar to parental tumors in immunodeficient mice [4]. Recently, the culture methods have diversified, and NSCs can be cultured *in vitro* as spheres or under adherent conditions in two-dimensional cultures or three-dimensional matrices. The sphere-forming assay was initially used to culture NSCs, which is considered to have limitations in terms of evaluating NSCs when removed from their *in vivo* environment [44]. Furthermore, the sphere-forming assay is not the only assay to isolate GSCs, because it has been suggested to induce distinct behavior of cells outside of their original environment.

Although CD133 is an important GSC marker [43], CD133(–) tumor cells isolated from GBMs also show stem-like cell properties [45, 46]. Recent studies have shown that CD133(–) cells can generate a CD133(+) cell population, suggesting that CD133(–) cancer cells might result in brain tumor initiation [47]. It is still unclear whether the expression of CD133 is associated with tumor initiation. These contradictory results suggest that GSCs may be heterogeneous and the diversity of GSCs might be one of the factors contributing to GBM heterogeneity. GSCs may not be a static population, but a dynamically modulated population because of genomic instability, differentiation, and plasticity. In fact, distinct GSCs have been isolated from the same GBM [48]. Furthermore, CSC plasticity has been reported in melanoma [49]. Therefore, a similar characteristic may be applicable to GSCs. There are still many unanswered questions regarding GSCs and further studies are necessary to improve our understanding.

### 4.1. Therapeutic Resistance of Glioma Stem Cells

Genetic mutations in target molecules of drugs, the expression of molecules that inactivate drugs, and mechanisms for excretion of drugs from cells have all been identified as ways that cancer cells acquire resistance to chemotherapeutic agents [50]. Some tumors that initially respond to chemotherapy subsequently recur, and the tumor cells in such cases are considered to have acquired drug resistance. However, after the existence of CSCs was demonstrated, and because CSCs are proficient at DNA repair and exhibit high gene expression levels of ATP-binding cassette half-transporter proteins, a new hypothesis has been proposed; CSCs are resistant to chemotherapeutic agents, and they persist after chemotherapy and become the source of tumor regrowth [50]. Many recent reports have shown that GSCs express multidrug resistance genes [51] and contribute to therapeutic resistance [52, 53]. It is now widely accepted that GSCs contribute to GBM recurrence after conventional therapies including chemotherapy and radiotherapy. In fact, GSCs are more resistant to radiation than non-GSCs [54]. In response to radiation-induced DNA damage, GSCs preferentially activate several critical DNA damage checkpoint proteins. As a result, GSCs are more efficient at repairing damaged DNA and recover more rapidly from DNA damage than non-GSCs. Although it has been reported that the postoperative first-line chemotherapy for malignant glioma, TMZ, preferentially removes GSCs expressing high levels of DNA repair protein MGMT [55], the drug does not inhibit self-renewal of GSCs expressing a normal level of MGMT [56].

As well as regulating stem cell proliferation and survival, the niche may also play a protective role of shielding stem cells from environmental insults [57]. It has been demonstrated that the vascular niche can protect GSCs from chemotherapy and radiotherapy [58]. VEGF is one of the best-characterized permeability factors that has been demonstrated to contribute to blood-brain barrier (BBB) breakdown in glioma directly [59]. Increased permeability of tumor blood vessels induced by VEGF results in elevated interstitial pressure and significant cerebral edema. The elevated interstitial pressure decreases the transport of drugs to tumor cells. Radiation therapy has also been demonstrated to induce VEGF expression in glioma cells [60]. Radiation-enhanced VEGF secretion is associated with an increased angiogenic potential of the tumor, which is thought to be a factor in radioresistance [61].

## 5. The Glioma Stem Cell Niche

Excessive and grossly disorganized blood vessel formation is a hallmark of GBM. This aberrant vascularity is presumed to be important for satisfying the nutritional demand of rapid tumor growth. NSCs within the subventricular zone and hippocampus are concentrated in regions that are rich in blood vessels, called the “vascular niche” [62]. This organization places the stem cells in a close relationship with endothelial and other vascular cells, which facilitates communication among these cell types. The vascular niche protects NSCs from apoptotic stimuli to maintain a good balance between self-renewal and differentiation. Similarly, GSCs are intimately associated with the vascular niche in tumors [63]. GSCs residing in the niche not only are protected from external factors but also maintain their stemness by receiving signals from the niche and thereby sustain tumor survival. Endothelial cells are one of the most important components in the vascular niche, which secrete paracrine factors that promote stem cell survival and self-renewal [64]. In the same manner, GSCs transplanted with endothelial cells grow more rapidly than when transplanted alone [63]. Thus, endothelial cells have been demonstrated to maintain the self-renewal capacity of GSCs. In addition, recent reports have suggested that GSCs can transdifferentiate into endothelial cells directly [65, 66]. Accumulating evidence has suggested that the functional relationship between GSCs and the vascular niche may be bidirectional in which GSCs may maintain the vascular niche and the vascular niche promotes proliferation and self-renewal of GSCs. 

A hypoxic environment is also a GSC niche that maintains their self-renewal [67, 68]. Hypoxia promotes expansion of the GSC fraction and regulates the expression of stem cell markers [69, 70]. Hypoxic conditions induce VEGF expression in both GSCs and non-GSCs, but the VEGF levels are consistently higher in GSCs [68]. High-level production of VEGF by GSCs can promote angiogenesis and their tumor-initiating capacity [71]. HIF-1 is a transcription factor that functions as a master regulator of oxygen homeostasis [72]. It has been reported that depletion of HIF in GSCs inhibits self-renewal, survival, and tumor initiation [68, 69]. In addition, HIF has an important role in upregulation of VEGF signaling and promotion of angiogenesis, resulting in maintenance of the tumor and its microenvironment.

## 6. Basic Concepts in Glioma Immunology

The concept of the “immune privileged” state of the central nervous system (CNS) was based on classical studies that showed that the brain is more permissive for transplantation of allografts than for areas outside the brain. Currently, general explanations for this concept include the absence of conventional lymphatics, the presence of the BBB, and the presumed paucity of antigen-presenting cells (APCs) within the neural parenchyma. However, our understanding of the immunoregulatory system of the CNS has expanded greatly over the past decade. Many of the participants of the immune system have been shown to play key roles in the CNS. In fact, immunocompetent cells have been demonstrated to exist within the brain parenchyma where they provide surveillance and permit the brain to mount an immunological response in association with the peripheral mechanisms of immunity, including both cellular and humoral components [73]. Furthermore, because activated T cells have been found to pass through the BBB and infiltrate the brain [74], various immunotherapeutic strategies to kill brain tumors are being attempted. 

### 6.1. Blood-Brain Barrier

The basic anatomical structure of the BBB consists of tight junctions that seal the margins of the endothelial cells of blood vessels in the CNS and severely impede diffusion of molecules from the blood. The BBB is maintained by glial cells, particularly astrocytes, which surround the vessels [75, 76]. Despite this apparent anatomical shield, activated lymphocytes as well as immunoglobulins and other serum proteins can cross the BBB according to their molecular weight and charge [77]. Under normal conditions, the endothelial cells of the BBB express very low levels of the adhesion molecules required for lymphocyte emigration. However, activation of the endothelial cells augments the passage of lymphocytes. Thus, the BBB is not an absolute barrier to all substances. 

### 6.2. T Cells

It is clear that T cells can enter the CNS in healthy animals [78]. Accumulating evidence indicates that T cells that have been activated to the blast phase can cross the BBB, whereas naïve or resting T cells do not gain entry [78–81]. If activated T cells can gain access to the CNS, T cells that are specific for neural antigens can theoretically produce CNS inflammation. However, spontaneous autoimmune diseases of the CNS are rare. These observations suggest that the CNS not only limits the entry of cells and antibodies but also provides a specific microenvironment that inhibits the function of immune cells. In fact, T cells in the CNS parenchyma are known to rapidly undergo apoptosis [82]. Fas/FasL-mediated mechanisms and the gangliosides of the brain have been suggested to be critical for controlling T cells in the CNS [82–85]. The concept that T cells “home to” the CNS does not have current support. No homing receptors have been identified, which specifically target any leukocyte type to the CNS endothelium. Rather, it has been suggested that activated T cells enter the CNS in a random manner, and upon encountering their antigen in the CNS, these cells can accumulate and elicit an immune reaction [79, 86].

### 6.3. Immunosuppression by Glioma

GBMs are profoundly immunosuppressive both locally and systemically. It is known that GBMs secrete multiple immunosuppressive factors, including transforming growth factor-*β* (TGF-*β*) and prostaglandin E2 (PGE2), which lead to the degradation of cellular immunity and abnormal immune cell activation [87]. TGF-*β* is known to expand the pool of immunosuppressive regulatory T cells, resulting in suppression of T cell proliferation. Both TGF-*β* and PGE2 downregulate the expression of major histocompatibility complex (MHC) class II, as well as the antigen processing of dendritic cells (DCs) [88]. Tumor-derived VEGF acts primarily as an angiogenic factor and has been shown to stimulate the proliferation of myeloid-derived suppressor cells that have significant immunosuppressive implications [89]. VEGF and interleukin- (IL-) 6 secreted from GBM activate signal transducer and activator of transcription that inhibits macrophage activation, induces an immunosuppressive macrophage phenotype (M2), and promotes GBM tumorigenesis [90–92]. Recently, it was demonstrated that GSCs express B7-H1 that induces T cell anergy and apoptosis to maintain the immunosuppressive environment called the “immune niche” [93]. Furthermore, it is increasingly evident that tumor-infiltrating monocytes/macrophages, circulating myeloid-derived suppressor cells, and regulatory T cells contribute significantly to both local and systemic immunosuppression in GBM patients [94]. Although the exact roles of these cells are still unclear, understanding and disrupting the immunosuppressive function of GBM may be essential for development of successful immunotherapeutic strategies. 

### 6.4. Glioma Antigens

Since the presence of tumor antigens was demonstrated in tumor cells, glioma antigens recognized by CTLs have also been identified, which are classified as cancer-testis (CT) antigens, tissue-specific antigens, mutated antigens, and others. CT antigens are expressed in a variety of human cancers, but not in normal tissues except for the testis, and represent promising targets for immunotherapeutic approaches [95]. MAGE-1 and SOX6 are both CT antigens and have been observed in gliomas but not in normal brain tissues [96, 97]. Tissue-specific antigens, such as gp100 and TRP-2, were originally identified as melanoma antigens and have been shown to be expressed in gliomas [98, 99]. The epidermal growth factor receptor (EGFR) is often amplified and structurally rearranged in malignant gliomas, and the most common mutation is the EGFRvIII mutated glioma antigen [7]. Other glioma antigens that have been identified include IL13Ra2 [100], EphA2 [101], EphB6 [102], AIM-2 [103], HER-2 [99], WT1 [104], ARF4L [105], SART-3 [106], SOX11 [107], KIF1C, and KIF3C [108]. Recently, the National Cancer Institute of the United States performed an in-depth review of 75 tumor-associated antigens that can be targeted by immunotherapy. The antigens were ranked according to nine factors: therapeutic function, immunogenicity, role in oncogenicity, specificity, expression level in tumors, stem cell expression, number of patients with positive tumors, number of antigenic epitopes, and cellular location of expression [109]. The highest ranked antigens included glioma antigens, WT1 and EGFRvIII, which are discussed in detail later in Section 7.3.2.

## 7. Immunotherapy for Glioma

As a result of the discovery of tumor antigen peptides that are recognized by T cells, it has become possible to artificially manipulate the immune response as a target of immunotherapy, which has allowed great advances to be made in tumor immunology [110]. Moreover, because T cells have been demonstrated to be important for tumor rejection, have the ability to proliferate specifically in response to tumors, and possess a memory mechanism, they play a central role in tumor immunotherapy. Immunotherapies consists of antibody-mediated immunotherapy, active immunotherapy that induces antitumor immunity in patients via a cancer vaccine, and adoptive (passive) immunotherapy in which tumor antigen-activated T cells are prepared *ex vivo* and administered to patients.

### 7.1. Antibody-Mediated Immunotherapy

Successful targeting of a specific antibody to a tumor is dependent on many factors including antigen stability, accessibility, and density within the tumor. Although antibodies generally do not cross the BBB, reduced integrity of the tight junctions between the capillary endothelial cells of the brain tumor neovasculature results in an increase of intratumoral permeability [111]. Early studies demonstrated the successful imaging of gliomas in glioma patients using radiolabeled antibodies [112], suggesting the feasibility of antibody-mediated immunotherapy of brain tumors. In addition, techniques for site-specific disruption of the BBB at the tumor site have been developed, which employ bradykinin agonists and focused ultrasound to allow chemotherapeutics or antibodies access to infiltrate tumor cells in the brain [113, 114]. 

One of the most extensively investigated glioma-associated antigens is tenascin, an extracellular matrix molecule that is prominently expressed in the fibrillary matrix and perivascular patterns of gliomas [115, 116]. Monoclonal antibodies (mAbs) specific for human tenascin have been generated, namely, BC-2 used in European clinical trials [117] and 81C6 used in clinical trials in the USA [118]. Malignant gliomas frequently overexpress the EGFR, and binding of the EGFR inhibits tumor growth and induces apoptosis in tumor cells [119]. Cetuximab (Erbitux) is a chimeric EGFR mAb that has shown effectiveness in preclinical studies, but a phase II trial with primary or recurrent GBM indicated no apparent therapeutic effect on progression-free survival (PFS) or overall survival (OS) [120]. Other EGFR mAbs, trastuzumab (Herceptin) and panitumumab (Vertibix), have been applied to numerous patients with breast and colon cancers, respectively, but little study of malignant glioma has been reported. An attractive alternative is targeting of the mutant-type EGFRvIII, an in-frame splice variant, which contributes to unchecked proliferation of gliomas [121]. A chimeric form of mAb ch806 has been administered to a patient with an anaplastic astrocytoma in a phase I study that showed localization of the mAb at the tumor [122]. The clinical efficacy of the EGFRvIII mAb has been evaluated further in recent clinical trials.

### 7.2. Adoptive Immunotherapy

Adoptive immunotherapy refers to cell transfer therapy in which the immune cells of the patient are manipulated *ex vivo* to specifically activate in response to glioma antigens and are subsequently retransferred to the patient. The use of T cells for the treatment of brain tumors has several advantages: (1) recognition of antigens by T cells is highly specific and can potentially be used to elicit a targeted response to tumor antigens, and (2) activated T cells have the capacity to traffic into brain tumors following systemic delivery. However, early clinical applications of adoptive T cell immunotherapy for brain tumors have been mostly ineffective, including lymphocyte-activated killer cells, which are autologous peripheral blood lymphocytes stimulated with IL-2 *in vitro* [123–129]. To improve the efficacy of conventional adoptive immunotherapy, the protocols have been modified. For example, malignant glioma patients were first vaccinated with autologous tumor cells and bacillus Calmette-Guerin as an adjuvant, and peripheral blood mononuclear cells (PBMCs) obtained from the patient were stimulated with IL-2 *ex vivo* and then reinfused into the patient [130]. However, no significant clinical responses were observed using this protocol. Another clinical trial was performed using systemic adoptive transfer of tumor-draining lymph node T cells that had been activated and expanded *ex vivo* [131]. In this protocol, glioma patients were vaccinated with irradiated autologous tumor cells admixed with granulocyte-macrophage colony-stimulating factor (GM-CSF). T cells were then isolated from the vaccine-draining lymph nodes and stimulated with the bacterial superantigen Staphylococcal enterotoxin A, and in some cases, further stimulated with an anti-CD3 antibody. In this clinical trial, 3/10 patients showed radiographic regression of recurrent malignant gliomas [131]. However, no study has proven prolongation of the OS of glioma patients. In addition, adoptive immunotherapy has some limitations such as difficulty in *ex vivo* cell expansion, and, more importantly, the antitumor effect is temporary because the transferred cells fail to engraft and persist. Subsequently, to overcome the problem of low survival of the transferred cells, combinatorial therapy with high-dose chemotherapy followed by adoptive immunotherapy has been developed, because chemotherapies may promote homeostatic proliferation/activation of transferred cells [132]. Furthermore, genetically modified T cells, which express a chimeric antigen receptor for tumor antigens, such as IL-13R*α*2 and HER2, have been reported to induce GBM regression in preclinical studies [133, 134]. Although adoptive immunotherapy has some issues, new strategies such as combinatorial therapy using high-dose chemotherapy have attracted clinical attention.

### 7.3. Active Immunotherapy

Active immunotherapy is cancer vaccine therapy that induces antitumor immunity in patients by vaccination using tumor antigens or modified tumor cells. Cancer vaccine therapy can be administered by various techniques including the use of DCs, various types of tumor cells and antigens (e.g., peptides, proteins, and genes), or in combination with adjuvants. Because peptides are biologically stable and easy to handle, clinical studies that have assessed antigen peptides represent the majority of research on immunotherapy of brain tumors [11, 135]. MHC class I-binding peptides, which are recognized by CTLs (CD8(+) T cells), and MHC class II-binding peptides, which are recognized by helper T cells (CD4(+) T cells), are among the tumor antigens recognized by T cells [136]. Because of technical reasons related to the identification of peptides, MHC class I-binding peptides with the ability to activate CTLs have mainly been used for the preparation of tumor vaccines. 

#### 7.3.1. Dendritic Cell-Based Cancer Vaccine

DCs are the most potent APCs and are the only cells able to prime naïve T cells. Although tumor cells present antigens, they are known to only weakly stimulate the CTL response *in vivo*. Possible explanations for this phenomenon include inefficient antigen presentation and a lack of costimulatory molecules to prime T cells. Therefore, professional APCs, namely, DCs, pulsed with tumor antigens are extensively being tested in cancer vaccine studies. Many preclinical studies have examined the effects of DCs loaded with different sources of tumor antigens including tumor extracts obtained by sonicating tumor cells, synthetic or acid-eluted peptides, tumor RNA or DNA, and DCs and tumor cells fused using a tension-active compound such as polyethylene glycol [137]. Furthermore, as single large-scale isolation and expansion of DCs in culture have become feasible, DC-based therapies have been successfully employed in several clinical trials for cancer such as melanoma [138], renal cell carcinoma [139], and prostate cancer [140]. Promising preclinical results of a DC-based cancer vaccine for glioma were also followed by clinical trials in glioma patients. 

A clinical trial using DCs pulsed with acid-eluted peptides from glioma tissue was associated with significantly increased survival. The median survival times for DC-treated and control groups were 455 and 257 days, respectively [141]. Peptides from the surface of cultured autologous tumor cells were acid-eluted and incubated with autologous DCs derived from patient PBMCs with IL-4 and GM-CSF. In addition, there was no occurrence of significant side effects or autoimmune toxicity. A clinical trial was performed with eight glioma patients who received DCs fused with autologous glioma cells by polyethylene glycol [142]. The clinical results included two partial responses (PRs) and no serious adverse effects. The most recent results of a phase I/II trial of a DC-based vaccine for 77 patients with newly diagnosed GBM also showed the feasibility of this treatment [143]. So far, DC-based cancer vaccines have been shown to be safe. However, at this point, it is difficult to determine the efficacy of DC vaccines because of the great differences in study designs that have been used. Therefore, the efficacy of DC vaccines remains to be more closely examined in randomized and controlled clinical trials.

#### 7.3.2. Peptide Vaccines

Peptide vaccination is the most promising immunotherapy and there are currently several ongoing clinical studies. In general, peptides used as cancer vaccines consist of nine amino acids capable of binding to MHC class I. APCs present these peptide antigens in association with MHC class I molecules, which activate CTLs that are reactive to tumor cells. Activated CTLs migrate into the brain and eliminate glioma cells. Although several clinical trials of peptide-based vaccines for various cancers have been reported, the responses are not significant [144]. In contrast, recent clinical trials of peptide-based vaccines for malignant gliomas have obtained promising results.

A targeted peptide vaccine against EGFRvIII, a mutated form of the EGFR, entered a phase I clinical trial, the Vaccine for Intra-Cranial Tumors I (VICTORI), and has continued to the phase II study, A Complementary Trial of an Immunotherapy Against Tumor Specific EGFRvIII (ACTIVATE) [145]. In the VICTORI phase I trial, patients with malignant glioma were administered vaccines in which DCs were pulsed with the EGFRvIII peptide conjugated to the adjuvant keyhole limpet hemocyanin (KLH). The median survival of GBM patients treated with vaccines was 22.8 months, which was longer than that of patients treated with TMZ (14.6 months) [146]. ACTIVATE, the following phase II trial, evaluated the efficacy of EGFRvIII peptide-KLH and GM-CSF without the use of DCs [7]. Patients with newly diagnosed GBM were enrolled in this trial and vaccinated monthly until evidence of tumor progression. The median time to progression (TTP) was 14.2 months and the median survival was 32 months, both of which were longer than those of the unvaccinated group. The follow-up phase II multicenter trial enrolled 21 patients with EGFRvIII-positive GBM, which were treated monthly with EGFRvIII peptide-KLH following standard surgical, radiation, and TMZ therapies until tumor progression. As a result, the median TTP was 16.6 months [8]. The EGFRvIII-targeted vaccine (CDX-10) is currently in an ongoing phase III randomized clinical trial and awaiting results. 

The WT1 gene was originally isolated as a gene responsible for Wilms' tumor, which is involved in cell proliferation and apoptosis [147]. Because the WT1 gene is overexpressed in various types of tumors including glioma, a phase II clinical trial for a WT1 peptide vaccine was conducted for 21 patients with recurrent GBM [11]. The clinical responses induced were two PRs and a median PFS of 5 months. At present, several clinical studies are ongoing.

Because the antigenicity of tumor antigens may be weak, cancer vaccines with a cocktail of tumor antigens have been developed and applied to GBM patients. In a clinical trial of vaccination with a peptide cocktail, patients who had exhibited a preexisting response to specific peptides were vaccinated with these peptides [9]. Using this strategy, faster and stronger activation of CTLs can be induced [148]. The results of a phase I study of vaccination with a peptide cocktail for 25 patients with malignant glioma have been reported [9]. Prevaccination PBMCs and plasma were collected to examine the patient response to the peptides. The most frequent reactive peptides were derived from squamous cell carcinoma antigen recognized by T cells 3, lymphocyte-specific protein tyrosine kinase, and multidrug resistance-associated protein 3. The clinical responses included five PRs and the median survival was 18 months. A recent clinical study of the personalized peptide cocktail vaccine for patients with GBM also reported promising results [10]. Thus, the peptide cocktail vaccine is a prospective concept for cancer vaccination and is currently in ongoing clinical trials.

## 8. Immunotherapy for Glioma Stem Cells

Conventional therapies including chemotherapy and radiotherapy provide only palliative effects on GBM, probably because they target proliferating nontumorigenic cells. In contrast, GSCs are considered mostly quiescent and are thus resistant to conventional therapies [53]. Therefore, more effective therapies are required for targeting GSCs, which are based on antitumor mechanism(s) that are different from those of conventional therapy. Recent findings suggest that immunotherapy is a promising strategy for targeting GSCs [16, 149, 150]. We have previously identified and analyzed glioma antigens using a variety of methods, based on their expression specificity and immunogenicity [151]. SOX6 was identified using SEREX (serological identification of antigens by recombinant expression cloning) [152] and may be a candidate antigen that targets GSCs because of its specific expression in glioma and GSCs. Human leukocyte antigen-A2- and A24-restricted CTL peptides derived from SOX6 have been identified, and glioma patient lymphocytes stimulated with these peptides are capable of inducing glioma-specific CTLs [16]. Moreover, it has been demonstrated that GSCs express SOX6 and are killed by CTLs primed against SOX6-derived peptides. Therefore, GSC antigen peptides recognized by CTLs can be applied to immunotherapy that targets GSCs. 

Another immunotherapeutic strategy targeting GSCs is DC-based vaccination with GSCs that are isolated and concentrated from glioma tissues. Recent preclinical studies have demonstrated the potential of DC vaccination using GSCs [13, 14]. In a mouse glioma model, DC vaccination using neurospheres of mouse GL261 glioma showed a stronger antitumor effect compared with that using adherent GL261 cells [13]. Although there were no data of tumor antigen characterization or other mechanistic data in this study, it indicated the distinct potential of GSCs to induce antitumor immunity. In another study with a rat glioma 9L model, DC vaccination using a lysate of 9L neurospheres, but not conventionally cultured 9L cells, induced CTLs that recognized GSCs and prolonged the survival of animals bearing 9L GSC tumors [14]. DC-based vaccination appears to have the potential to be an effective treatment for malignant gliomas, and autologous GSCs isolated from glioma patients can be applied clinically at an early stage. 

## 9. Immunotherapy Targeting the Tumor Microenvironment

GSCs and the vascular niche have a bidirectional relationship. GSC properties are maintained by the vascular niche and the vascular niche is maintained by VEGF secreted from GSCs. VEGFs/VEGFRs are the major therapeutic targets to disrupt this interaction. Bevacizumab (Avastin), a humanized monoclonal antibody that binds to VEGF, prevents VEGF from binding to its receptors and activating signaling cascades that lead to angiogenesis [153]. It has been demonstrated that bevacizumab can disrupt the vascular niche in which GSCs reside [53]. Recent clinical trials demonstrated the benefit of bevacizumab for the treatment of recurrent GBM by a reduction of peritumoral cerebral edema and a prolonged PFS [153–156]. A phase II trial has been performed to evaluate the efficacy of bevacizumab in combination with chemotherapy (irinotecan) for patients with recurrent high-grade glioma [153]. Twenty of the 35 patients had at least a PR and the 6-month PFS for treated patients was 46%, which is significantly better than historical controls (generally less than 30%). A recent multicenter phase II study for newly diagnosed GBM also showed that bevacizumab combined with radiotherapy and TMZ improves PFS, but not OS, compared with that of the control group. 

Another immunotherapeutic strategy for targeting the vascular niche is vaccines targeting VEGFRs [157, 158]. Targeting tumor angiogenesis by vaccines has potential advantages over targeting tumor cells. First, tumor endothelial cells are more accessible to the immune system than that of tumor cells at a distance from vessels [159]. Tumor endothelial cells are readily accessed by lymphocytes in the bloodstream, and CTLs can directly damage endothelia cells without penetration of any other tissue type. Second, the loss or downregulation of MHC molecules on tumor cells is considered to be one of the major reasons for the limited clinical efficacy of cancer vaccines [160]. Because such MHC loss has not been reported for the endothelial cells of newly formed vessels in tumors, the development of vaccines against vascular endothelial cells in tumors may overcome the immunological evasion of tumors. A phase I clinical trial using a peptide vaccine against VFGFR-2 in combination with gemcitabine has been performed for patients with advanced pancreatic cancer, and peptide-specific CTLs were found to be induced [158]. Another phase I clinical trial using a peptide vaccine against VEGFR-1 was performed for advanced solid tumors, and activation of peptide-specific CTLs was also demonstrated [157]. Although clinical study of VEGFR peptide vaccines has just begun and further clinical studies would be essential to demonstrate their clinical benefits, this strategy alone or in combination with tumor vaccines is an alternative and promising immunotherapy for targeting the vascular niche. A recent case report of peptide cocktail vaccines consisting of three tumor antigen peptides and two VEGFR peptides (VEGFR-1 and VEGFR-2) showed significant tumor shrinkage in a patient with advanced colon cancer [161]. Since the use of antiangiogenic drugs such as bevacizumab has become widespread, it has been found that tumors develop mechanisms of resistance to antiangiogenic drugs. Therefore, multimodal therapies including combinations of antiangiogenic therapies together with cytotoxic therapies should be considered to overcome this problem.

## 10. Conclusions

GSCs have an extraordinary capacity for tumor initiation and are highly resistant to conventional therapies. Therefore, an optimal therapeutic strategy for treating GBM should be directed against GSCs. Based on the results of recent studies, immunity can be modulated and controlled in the brain, similar to that in other organs. Activated T cells are capable of entering the brain across the BBB, and selectively attack GSCs. Recent clinical studies indicate that immunotherapies show promise as an effective method of targeting both GSCs and their niche for the treatment of GBM. However, GBM is one of the most aggressive cancers, and further development is required for multimodal treatment methods that use combinations of different cytotoxic mechanisms that target GSCs and their niche.

## References

[1] Kleihues P, Louis DN, Scheithauer BW (2002). The WHO classification of tumors of the nervous system. *Journal of Neuropathology and Experimental Neurology*.

[2] Bonnet D, Dick JE (1997). Human acute myeloid leukemia is organized as a hierarchy that originates from a primitive hematopoietic cell. *Nature Medicine*.

[3] Singh SK, Clarke ID, Terasaki M (2003). Identification of a cancer stem cell in human brain tumors. *Cancer Research*.

[4] Lee J, Kotliarova S, Kotliarov Y (2006). Tumor stem cells derived from glioblastomas cultured in bFGF and EGF more closely mirror the phenotype and genotype of primary tumors than do serum-cultured cell lines. *Cancer Cell*.

[5] Chen J, McKay RM, Parada LF (2012). Malignant glioma: lessons from genomics, mouse models, and stem cells. *Cell*.

[6] Filatova A, Acker T, Garvalov BK (2013). The cancer stem cell niche(s): the crosstalk between glioma stem cells and their microenvironment. *Biochimica et Biophysica Acta*.

[7] Heimberger AB, Crotty LE, Archer GE (2003). Epidermal growth factor receptor VIII peptide vaccination is efficacious against established intracerebral tumors. *Clinical Cancer Research*.

[8] Sampson JH, Heimberger AB, Archer GE (2010). Immunologic escape after prolonged progression-free survival with epidermal growth factor receptor variant III peptide vaccination in patients with newly diagnosed glioblastoma. *Journal of Clinical Oncology*.

[9] Yajima N, Yamanaka R, Mine T (2005). Immunologic evaluation of personalized peptide vaccination for patients with advanced malignant glioma. *Clinical Cancer Research*.

[10] Terasaki M, Shibui S, Narita Y (2011). Phase I trial of a personalized peptide vaccine for patients positive for human leukocyte antigen-A24 with recurrent or progressive glioblastoma multiforme. *Journal of Clinical Oncology*.

[11] Izumoto S, Tsuboi A, Oka Y (2008). Phase II clinical trial of Wilms tumor 1 peptide vaccination for patients with recurrent glioblastoma multiforme. *Journal of Neurosurgery*.

[12] Iwami K, Shimato S, Ohno M (2012). Peptide-pulsed dendritic cell vaccination targeting interleukin-13 receptor *α*2 chain in recurrent malignant glioma patients with HLA-A*24/A*02 allele. *Cytotherapy*.

[13] Pellegatta S, Poliani PL, Corno D (2006). Neurospheres enriched in cancer stem-like cells are highly effective in eliciting a dendritic cell-mediated immune response against malignant gliomas. *Cancer Research*.

[14] Xu Q, Liu G, Yuan X (2009). Antigen-specific T-cell response from dendritic cell vaccination using cancer stem-like cell-associated antigens. *Stem Cells*.

[15] Schmitz M, Temme A, Senner V (2007). Identification of SOX2 as a novel glioma-associated antigen and potential target for T cell-based immunotherapy. *The British Journal of Cancer*.

[16] Ueda R, Ohkusu-Tsukada K, Fusaki N (2010). Identification of HLA-A2- And A24-restricted T-cell epitopes derived from SOX6 expressed in glioma stem cells for immunotherapy. *International Journal of Cancer*.

[17] Louis DN, Ohgaki H, Wiestler OD (2007). The 2007 WHO classification of tumours of the central nervous system. *Acta Neuropathologica*.

[18] Parsons DW, Jones S, Zhang X (2008). An integrated genomic analysis of human glioblastoma multiforme. *Science*.

[19] McLendon R, Friedman A, Bigner D (2008). Comprehensive genomic characterization defines human glioblastoma genes and core pathways. *Nature*.

[20] Kleihues P, Ohgaki H (1999). Primary and secondary glioblastomas: from concept to clinical diagnosis. *Neuro-Oncology*.

[21] Ohgaki H, Kleihues P (2007). Genetic pathways to primary and secondary glioblastoma. *The American Journal of Pathology*.

[22] Maher EA, Brennan C, Wen PY (2006). Marked genomic differences characterize primary and secondary glioblastoma subtypes and identify two distinct molecular and clinical secondary glioblastoma entities. *Cancer Research*.

[23] Tso CL, Freije WA, Day A (2006). Distinct transcription profiles of primary and secondary glioblastoma subgroups. *Cancer Research*.

[24] Yan H, Parsons DW, Jin G (2009). IDH1 and IDH2 mutations in gliomas. *The New England Journal of Medicine*.

[25] Noushmehr H, Weisenberger DJ, Diefes K (2010). Identification of a CpG island methylator phenotype that defines a distinct subgroup of glioma. *Cancer Cell*.

[26] Gerson SL (2002). Clinical relevance of MGMT in the treatment of cancer. *Journal of Clinical Oncology*.

[27] Hegi ME, Diserens AC, Gorlia T (2005). MGMT gene silencing and benefit from temozolomide in glioblastoma. *The New England Journal of Medicine*.

[28] Rafii S, Lyden D, Benezra R, Hattori K, Heissig B (2002). Vascular and haematopoietic stem cells: novel targets for anti-angiogenesis therapy?. *Nature Reviews Cancer*.

[29] Semenza GL (2010). Defining the role of hypoxia-inducible factor 1 in cancer biology and therapeutics. *Oncogene*.

[30] Rong Y, Durden DL, Van Meir EG, Brat DJ (2006). ‘Pseudopalisading’ necrosis in glioblastoma: a familiar morphologic feature that links vascular pathology, hypoxia, and angiogenesis. *Journal of Neuropathology and Experimental Neurology*.

[31] Heddleston JM, Li Z, Lathia JD, Bao S, Hjelmeland AB, Rich JN (2010). Hypoxia inducible factors in cancer stem cells. *The British Journal of Cancer*.

[32] Kowanetz M, Ferrara N (2006). Vascular endothelial growth factor signaling pathways: therapeutic perspective. *Clinical Cancer Research*.

[33] Sullivan LA, Brekken RA (2010). The VEGF family in cancer and antibody-based strategies for their inhibition. *MAbs*.

[34] Carmeliet P, Moons L, Luttun A (2001). Synergism between vascular endothelial growth factor and placental growth factor contributes to angiogenesis and plasma extravasation in pathological conditions. *Nature Medicine*.

[35] Hicklin DJ, Ellis LM (2005). Role of the vascular endothelial growth factor pathway in tumor growth and angiogenesis. *Journal of Clinical Oncology*.

[36] Grau SJ, Trillsch F, Herms J (2007). Expression of VEGFR3 in glioma endothelium correlates with tumor grade. *Journal of Neuro-Oncology*.

[37] Kaplan RN, Riba RD, Zacharoulis S (2005). VEGFR1-positive haematopoietic bone marrow progenitors initiate the pre-metastatic niche. *Nature*.

[38] Knizetova P, Ehrmann J, Hlobilkova A (2008). Autocrine regulation of glioblastoma cell cycle progression, viability and radioresistance through the VEGF-VEGFR2 (KDR) interplay. *Cell Cycle*.

[39] Dias S, Hattori K, Zhu Z (2000). Autocrine stimulation of VEGFR-2 activates human leukemic cell growth and migration. *Journal of Clinical Investigation*.

[40] Jenny B, Harrison JA, Baetens D (2006). Expression and localization of VEGF-C and VEGFR-3 in glioblastomas and haemangioblastomas. *Journal of Pathology*.

[41] Clarke MF, Dick JE, Dirks PB (2006). Cancer stem cells—perspectives on current status and future directions: AACR workshop on cancer stem cells. *Cancer Research*.

[42] Vescovi AL, Galli R, Reynolds BA (2006). Brain tumour stem cells. *Nature Reviews Cancer*.

[43] Singh SK, Hawkins C, Clarke ID (2004). Identification of human brain tumour initiating cells. *Nature*.

[44] Pastrana E, Silva-Vargas V, Doetsch F (2011). Eyes wide open: a critical review of sphere-formation as an assay for stem cells. *Cell Stem Cell*.

[45] Beier D, Hau P, Proescholdt M (2007). CD133^+^ and CD133^−^ glioblastoma-derived cancer stem cells show differential growth characteristics and molecular profiles. *Cancer Research*.

[46] Joo KM, Kim SY, Jin X (2008). Clinical and biological implications of CD133-positive and CD133-negative cells in glioblastomas. *Laboratory Investigation*.

[47] Chen R, Nishimura MC, Bumbaca SM (2010). A Hierarchy of self-renewing tumor-initiating cell types in glioblastoma. *Cancer Cell*.

[48] Piccirillo SGM, Combi R, Cajola L (2009). Distinct pools of cancer stem-like cells coexist within human glioblastomas and display different tumorigenicity and independent genomic evolution. *Oncogene*.

[49] Roesch A, Fukunaga-Kalabis M, Schmidt EC (2010). A temporarily distinct subpopulation of slow-cycling melanoma cells is required for continuous tumor growth. *Cell*.

[50] Dean M, Fojo T, Bates S (2005). Tumour stem cells and drug resistance. *Nature Reviews Cancer*.

[51] Salmaggi A, Boiardi A, Gelati M (2006). Glioblastoma-derived tumorospheres identify a population of tumor stem-like cells with angiogenic potential and enhanced multidrug resistance phenotype. *Glia*.

[52] Kang MK, Kang SK (2007). Tumorigenesis of chemotherapeutic drug-resistant cancer stem-like cells in brain glioma. *Stem Cells and Development*.

[53] Persano L, Rampazzo E, Basso G, Viola G (2013). Glioblastoma cancer stem cells: role of the microenvironment and therapeutic targeting. *Biochemical Pharmacology*.

[54] Bao S, Wu Q, McLendon RE (2006). Glioma stem cells promote radioresistance by preferential activation of the DNA damage response. *Nature*.

[55] Beier D, Röhrl S, Pillai DR (2008). Temozolomide preferentially depletes cancer stem cells in glioblastoma. *Cancer Research*.

[56] Clement V, Sanchez P, de Tribolet N, Radovanovic I, Ruiz i Altaba A (2007). HEDGEHOG-GLI1 signaling regulates human glioma growth, cancer stem cell self-renewal, and tumorigenicity. *Current Biology*.

[57] Moore KA, Lemischka IR (2006). Stem cells and their niches. *Science*.

[58] Huang Z, Cheng L, Guryanova OA, Wu Q, Bao S (2010). Cancer stem cells in glioblastoma-molecular signaling and therapeutic targeting. *Protein and Cell*.

[59] Tate MC, Aghi MK (2009). Biology of angiogenesis and invasion in glioma. *Neurotherapeutics*.

[60] Hovinga KE, Stalpers LJA, van Bree C (2005). Radiation-enhanced vascular endothelial growth factor (VEGF) secretion in glioblastoma multiforme cell lines—a clue to radioresistance?. *Journal of Neuro-Oncology*.

[61] Gorski DH, Beckett MA, Jaskowiak NT (1999). Blockade of the vascular endothelial growth factor stress response increases the antitumor effects of ionizing radiation. *Cancer Research*.

[62] Palmer TD, Willhoite AR, Gage FH (2000). Vascular niche for adult hippocampal neurogenesis. *The Journal of Comparative Neurology*.

[63] Calabrese C, Poppleton H, Kocak M (2007). A perivascular niche for brain tumor stem cells. *Cancer Cell*.

[64] Shen Q, Goderie SK, Jin L (2004). Endothelial cells stimulate self-renewal and expand neurogenesis of neural stem cells. *Science*.

[65] Ricci-Vitiani L, Pallini R, Biffoni M (2010). Tumour vascularization via endothelial differentiation of glioblastoma stem-like cells. *Nature*.

[66] Wang R, Chadalavada K, Wilshire J (2010). Glioblastoma stem-like cells give rise to tumour endothelium. *Nature*.

[67] Clarke L, Van Der Kooy D (2009). Low oxygen enhances primitive and definitive neural stem cell colony formation by inhibiting distinct cell death pathways. *Stem Cells*.

[68] Li Z, Bao S, Wu Q (2009). Hypoxia-inducible factors regulate tumorigenic capacity of glioma stem cells. *Cancer Cell*.

[69] Heddleston JM, Li Z, McLendon RE, Hjelmeland AB, Rich JN (2009). The hypoxic microenvironment maintains glioblastoma stem cells and promotes reprogramming towards a cancer stem cell phenotype. *Cell Cycle*.

[70] McCord AM, Jamal M, Shankavarum UT, Lang FF, Camphausen K, Tofilon PJ (2009). Physiologic oxygen concentration enhances the stem-like properties of CD133^+^ human glioblastoma cells *in vitro*. *Molecular Cancer Research*.

[71] Bao S, Wu Q, Sathornsumetee S (2006). Stem cell-like glioma cells promote tumor angiogenesis through vascular endothelial growth factor. *Cancer Research*.

[72] Ferrara N, Gerber HP, LeCouter J (2003). The biology of VEGF and its receptors. *Nature Medicine*.

[73] Hickey WF (2001). Basic principles of immunological surveillance of the normal central nervous system. *Glia*.

[74] Becher B, Prat A, Antel JP (2000). Brain-immune connection: immuno-regulatory properties of CNS-resident cells. *Glia*.

[75] Kuchler-Bopp S, Delaunoy JP, Artault JC, Zaepfel M, Dietrich JB (1999). Astrocytes induce several blood-brain barrier properties in non-neural endothelial cells. *NeuroReport*.

[76] Akiyama H, Kondoh T, Kokunai T, Nagashima T, Saito N, Tamaki N (2000). Blood-brain barrier formation of grafted human umbilical vein endothelial cells in athymic mouse brain. *Brain Research*.

[77] Bart J, Groen HJM, Hendrikse NH, van der Graaf WTA, Vaalburg W, de Vries EGE (2000). The blood-brain barrier and oncology: new insights into function and modulation. *Cancer Treatment Reviews*.

[78] Hickey WF (1999). Leukocyte traffic in the central nervous system: the participants and their roles. *Seminars in Immunology*.

[79] Flügel A, Willem M, Berkowicz T, Wekerle H (1999). Gene transfer into CD4^+^ T lymphocytes: green fluorescent protein- engineered, encephalitogenic T cells illuminate brain autoimmune responses. *Nature Medicine*.

[80] Fritz RB, Wang X, Zhao ML (2000). The fate of adoptively transferred quiescent encephalitogenic T cells in normal and antigen-tolerized mice. *Journal of Neuroimmunology*.

[81] Kerschensteiner M, Gallmeier E, Behrens L (1999). Activated human T cells, B cells, and monocytes produce brain-derived neurotrophic factor *in vitro* and in inflammatory brain lesions: a neuroprotective role of inflammation?. *Journal of Experimental Medicine*.

[82] Bauer J, Bradl M, Hickey WF (1998). T-cell apoptosis in inflammatory brain lesions: destruction of T cells does not depend on antigen recognition. *The American Journal of Pathology*.

[83] Flügel A, Schwaiger FW, Neumann H (2000). Neuronal FasL induces cell death of encephalitogenic T lymphocytes. *Brain Pathology*.

[84] Suvannavejh GC, Dal Canto MC, Matis LA, Miller SD (2000). Fas-mediated apoptosis in clinical remissions of relapsing experimental autoimmune encephalomyelitis. *Journal of Clinical Investigation*.

[85] Irani DN (1998). Brain-derived gangliosides induce cell cycle arrest in a murine T cell line. *Journal of Neuroimmunology*.

[86] Qing Z, Sewell D, Sandor M, Fabry Z (2000). Antigen-specific T cell trafficking into the central nervous system. *Journal of Neuroimmunology*.

[87] Albesiano E, Han JE, Lim M (2010). Mechanisms of Local Immunoresistance in Glioma. *Neurosurgery Clinics of North America*.

[88] Facoetti A, Nano R, Zelini P (2005). Human leukocyte antigen and antigen processing machinery component defects in astrocytic tumors. *Clinical Cancer Research*.

[89] Gabrilovich D (2004). Mechanisms and functional significance of tumour-induced dendritic-cell defects. *Nature Reviews Immunology*.

[90] Lang R, Patel D, Morris JJ, Rutschman RL, Murray PJ (2002). Shaping gene expression in activated and resting primary macrophages by IL-10. *Journal of Immunology*.

[91] Mancino A, Lawrence T (2010). Nuclear factor-*κ*B and tumor-associated macrophages. *Clinical Cancer Research*.

[92] Brantley EC, Benveniste EN (2008). Signal transducer and activator of transcription-3: a molecular hub for signaling pathways in gliomas. *Molecular Cancer Research*.

[93] di Tomaso T, Mazzoleni S, Wang E (2010). Immunobiological characterization of cancer stem cells isolated from glioblastoma patients. *Clinical Cancer Research*.

[94] Gustafson MP, Lin Y, New KC (2010). Systemic immune suppression in glioblastoma: the interplay between CD14^+^HLA-DR^lo/neg^ monocytes, tumor factors, and dexamethasone. *Neuro-Oncology*.

[95] Chen YT, Scanlan MJ, Sahin U (1997). A testicular antigen aberrantly expressed in human cancers detected by autologous antibody screening. *Proceedings of the National Academy of Sciences of the United States of America*.

[96] Kuramoto T (1997). Detection of MAGE-1 tumor antigen in brain tumor. *Kurume Medical Journal*.

[97] Ueda R, Yoshida K, Kawakami Y, Kawase T, Toda M (2004). Expression of a transcriptional factor, SOX6, in human gliomas. *Brain Tumor Pathology*.

[98] Chi DDJ, Merchant RE, Rand R (1997). Molecular detection of tumor-associated antigens shared by human cutaneous melanomas and gliomas. *The American Journal of Pathology*.

[99] Liu G, Ying H, Zeng G, Wheeler CJ, Black KL, Yu JS (2004). HER-2, gp100, and MAGE-1 are expressed in human glioblastoma and recognized by cytotoxic T cells. *Cancer Research*.

[100] Okano F, Storkus WJ, Chambers WH, Pollack IF, Okada H (2002). Identification of a novel HLA-A∗0201-restricted, cytotoxic T lymphocyte epitope in a human glioma-associated antigen, interleukin 13 receptor *α*2 chain. *Clinical Cancer Research*.

[101] Hatano M, Eguchi J, Tatsumi T (2005). EphA2 as a glioma-associated antigen: a novel target for glioma vaccines. *Neoplasia*.

[102] Jin M, Komohara Y, Shichijo S (2008). Identification of EphB6 variant-derived epitope peptides recognized by cytotoxic T-lymphocytes from HLA-A24+ malignant glioma patients. *Oncology Reports*.

[103] Liu G, Yu JS, Zeng G (2004). AIM-2: a novel tumor antigen is expressed and presented by human glioma cells. *Journal of Immunotherapy*.

[104] Hashiba T, Izumoto S, Kagawa N (2007). Expression of WT1 protein and correlation with cellular proliferation in glial tumors. *Neurologia Medico-Chirurgica*.

[105] Nonaka Y, Tsuda N, Shichijo S (2002). Recognition of ADP-ribosylation factor 4-like by HLA-A2-restricted and tumor-reactive cytotoxic T lymphocytes from patients with brain tumors. *Tissue Antigens*.

[106] Murayama K, Kobayashi T, Imaizumi T (2000). Expression of the SART3 tumor-rejection antigen in brain tumors and induction of cytotoxic T lymphocytes by its peptides. *Journal of Immunotherapy*.

[107] Schmitz M, Wehner R, Stevanovic S (2007). Identification of a naturally processed T cell epitope derived from the glioma-associated protein SOX11. *Cancer Letters*.

[108] Harada M, Ishihara Y, Itoh K, Yamanaka R (2007). Kinesin superfamily protein-derived peptides with the ability to induce glioma-reactive cytotoxic T lymphocytes in human leukocyte antigen-A24+ glioma patients. *Oncology Reports*.

[109] Cheever MA, Allison JP, Ferris AS (2009). The prioritization of cancer antigens: a national cancer institute pilot project for the acceleration of translational research. *Clinical Cancer Research*.

[110] Rosenberg SA (1999). A new era for cancer immunotherapy based on the genes that encode cancer antigens. *Immunity*.

[111] Fuchs HE, Archer GE, Colvin OM (1990). Activity of intrathecal 4-hydroperoxycyclophosphamide in a nude rat model of human neoplastic meningitis. *Cancer Research*.

[112] Day ED, Lassiter S, Woodhall B, Mahaley JL, Mahaley MS (1965). The localization of radioantibodies in human brain tumors. I. Preliminary exploration. *Cancer Research*.

[113] Bidros DS, Vogelbaum MA (2009). Novel drug delivery strategies in neuro-oncology. *Neurotherapeutics*.

[114] de Vries NA, Beijnen JH, Boogerd W, Van Tellingen O (2006). Blood-brain barrier and chemotherapeutic treatment of brain tumors. *Expert Review of Neurotherapeutics*.

[115] Bigner DD, Brown M, Coleman RE (1995). Phase I studies of treatment of malignant gliomas and neoplastic meningitis with 131I-radiolabeled monoclonal antibodies anti-tenascin 81C6 and anti-chondroitin proteoglycan sulfate Me1-14 F(ab’)2—a preliminary report. *Journal of Neuro-Oncology*.

[116] Kurpad SN, Zhao XG, Wikstrand CJ, Batra SK, McLendon RE, Bigner DD (1995). Tumor antigens in astrocytic gliomas. *Glia*.

[117] Riva P, Arista A, Franceschi G (1995). Local treatment of malignant gliomas by direct infusion of specific monoclonal antibodies labeled with131I: comparison of the results obtained in recurrent and newly diagnosed tumors. *Cancer Research*.

[118] Murphy-Ullrich JE, Lightner VA, Aukhil I, Yan YZ, Erickson HP, Hook M (1991). Focal adhesion integrity is downregulated by the alternatively spliced domain of human tenascin. *Journal of Cell Biology*.

[119] Eller JL, Longo SL, Hicklin DJ (2002). Activity of anti-epidermal growth factor receptor monoclonal antibody C225 against glioblastoma multiforme. *Neurosurgery*.

[120] Neyns B, Sadones J, Joosens E (2009). Stratified phase II trial of cetuximab in patients with recurrent high-grade glioma. *Annals of Oncology*.

[121] Wikstrand CJ, McLendon RE, Friedman AH, Bigner DD (1997). Cell surface localization and density of the tumor-associated variant of the epidermal growth factor receptor, EGFRvIII. *Cancer Research*.

[122] Scott AM, Lee FT, Tebbutt N (2007). Erratum: a phase I clinical trial with monoclonal antibody ch806 targeting transitional state and mutant epidermal growth factor receptors. *Proceedings of the National Academy of Sciences of the United States of America*.

[123] Jacobs SK, Wilson DJ, Kornblith PL, Grimm EA (1986). Interleukin-2 and autologous lymphokine-activated killer cells in the treatment of malignant glioma. Preliminary report. *Journal of Neurosurgery*.

[124] Yoshida S, Tanaka R, Takai N, Ono K (1988). Local administration of autologous lymphokine-activated killer cells and recombinant interleukin 2 to patients with malignant brain tumors. *Cancer Research*.

[125] Merchant RE, Merchant LH, Cook SHS, McVicar DW, Young HF (1988). Intralesional infusion of lymphokine-activated killer (LAK) cells and recombinant Interleukin-2 (rIL-2) for the treatment of patients with malignant brain tumor. *Neurosurgery*.

[126] Barba D, Saris SC, Holder C, Rosenberg SA, Oldfield EH (1989). Intratumoral LAK cell and interleukin-2 therapy of human gliomas. *Journal of Neurosurgery*.

[127] Boiardi A, Silvani A, Ruffini PA (1994). Loco-regional immunotherapy with recombinant interleukin-2 and adherent lymphokine-activated killer cells (A-LAK) in recurrent glioblastoma patients. *Cancer Immunology Immunotherapy*.

[128] Kruse CA, Schiltz PM, Bellgrau D, Kong Q, Kleinschmidt-DeMasters BK (1994). Intracranial administrations of single or multiple source allogeneic cytotoxic T lymphocytes: chronic therapy for primary brain tumors. *Journal of Neuro-Oncology*.

[129] Hayes RL, Koslow M, Hiesiger EM (1995). Improved long term survival after intracavitary interleukin-2 and lymphokine-activated killer cells for adults with recurrent malignant glioma. *Cancer*.

[130] Holladay FP, Heitz-Turner T, Bayer WL, Wood GW (1996). Autologous tumor cell vaccination combined with adoptive cellular immunotherapy in patients with Grade III/IV astrocytoma. *Journal of Neuro-Oncology*.

[131] Plautz GE, Barnett GH, Miller DW (1998). Systemic T cell adoptive immunotherapy of malignant gliomas. *Journal of Neurosurgery*.

[132] Bracci L, Moschella F, Sestili P (2007). Cyclophosphamide enhances the antitumor efficacy of adoptively transferred immune cells through the induction of cytokine expression, B-cell and T-cell homeostatic proliferation, and specific tumor infiltration. *Clinical Cancer Research*.

[133] Kahlon KS, Brown C, Cooper LJN, Raubitschek A, Forman SJ, Jensen MC (2004). Specific recognition and killing of glioblastoma multiforme by interleukin 13-zetakine redirected cytolytic T cells. *Cancer Research*.

[134] Ahmed N, Salsman VS, Kew Y (2010). HER2-specific T cells target primary glioblastoma stem cells and induce regression of autologous experimental tumors. *Clinical Cancer Research*.

[135] Yamanaka R (2008). Cell- and peptide-based immunotherapeutic approaches for glioma. *Trends in Molecular Medicine*.

[136] Kawakami Y, Rosenberg SA (1997). Human tumor antigens recognized by T-cells. *Immunologic Research*.

[137] Gong J, Chen D, Kashiwaba M, Kufe D (1997). Induction of antitumor activity by immunization with fusions of dendritic and carcinoma cells. *Nature Medicine*.

[138] Thurner B, Haendle I, Röder C (1999). Vaccination with Mage-3A1 peptide-pulsed nature, monocyte-derived dendritic cells expands specific cytotoxic T cells and induces regression of some metastases in advanced stage IV melanoma. *Journal of Experimental Medicine*.

[139] Kugler A, Stuhler G, Walden P (2000). Regression of human metastatic renal cell carcinoma after vaccination with tumor cell-dendritic cell hybrids. *Nature Medicine*.

[140] Lodge PA, Jones LA, Bader RA, Murphy GP, Salgaller ML (2000). Dendritic cell-based immunotherapy of prostate cancer: immune monitoring of a phase II clinical trial. *Cancer Research*.

[141] Yu JS, Wheeler CJ, Zeltzer PM (2001). Vaccination of malignant glioma patients with peptide-pulsed dendritic cells elicits systemic cytotoxicity and intracranial T-cell infiltration. *Cancer Research*.

[142] Kikuchi T, Akasaki Y, Irie M, Homma S, Abe T, Ohno T (2001). Results of a phase I clinical trial of vaccination of glioma patients with fusions of dendritic and glioma cells. *Cancer Immunology, Immunotherapy*.

[143] Ardon H, van Gool SW, Verschuere T (2012). Integration of autologous dendritic cell-based immunotherapy in the standard of care treatment for patients with newly diagnosed glioblastoma: results of the HGG-2006 phase I/II trial. *Cancer Immunol Immunother*.

[144] Rosenberg SA, Yang JC, Restifo NP (2004). Cancer immunotherapy: moving beyond current vaccines. *Nature Medicine*.

[145] Sampson JH, Archer GE, Mitchell DA, Heimberger AB, Bigner DD (2008). Tumor-specific immunotherapy targeting the EGFRvIII mutation in patients with malignant glioma. *Seminars in Immunology*.

[146] Stupp R, Mason WP, van den Bent MJ (2005). Radiotherapy plus concomitant and adjuvant temozolomide for glioblastoma. *The New England Journal of Medicine*.

[147] Oka Y, Tsuboi A, Elisseeva OA, Udaka K, Sugiyama H (2002). WT1 as a novel target antigen for cancer immunotherapy. *Current Cancer Drug Targets*.

[148] Mine T, Sato Y, Noguchi M (2004). Humoral responses to peptides correlate with overall survival in advanced cancer patients vaccinated with peptides based on pre-existing, peptide-specific cellular responses. *Clinical Cancer Research*.

[149] Xu Q, Yuan X, Yu JS (2012). Glioma stem cell research for the development of immunotherapy. *Advances in Experimental Medicine and Biology*.

[150] Li Z, Lee JW, Mukherjee D (2012). Immunotherapy targeting glioma stem cells—insights and perspectives. *Expert Opinion on Biological Therapy*.

[151] Toda M (2004). Cancer vaccine for brain tumors and brain tumor antigens. *Cancer Therapy*.

[152] Ueda R, Iizuka Y, Yoshida K, Kawase T, Kawakami Y, Toda M (2004). Identification of a human glioma antigen, SOX6, recognized by patients’ sera. *Oncogene*.

[153] Vredenburgh JJ, Desjardins A, Herndon JE (2007). Bevacizumab plus irinotecan in recurrent glioblastoma multiforme. *Journal of Clinical Oncology*.

[154] Lai A, Tran A, Nghiemphu PL (2011). Phase II study of bevacizumab plus temozolomide during and after radiation therapy for patients with newly diagnosed glioblastoma multiforme. *Journal of Clinical Oncology*.

[155] Chamberlain MC, Johnston SK (2010). Salvage therapy with single agent bevacizumab for recurrent glioblastoma. *Journal of Neuro-Oncology*.

[156] Norden AD, Young GS, Setayesh K (2008). Bevacizumab for recurrent malignant gliomas: efficacy, toxicity, and patterns of recurrence. *Neurology*.

[157] Hayashi H, Kurata T, Fujisaka Y (2013). Phase I trial of OTS11101, an anti-angiogenic vaccine targeting vascular endothelial growth factor receptor 1 in solid tumor. *Cancer Science*.

[158] Miyazawa M, Ohsawa R, Tsunoda T (2010). Phase I clinical trial using peptide vaccine for human vascular endothelial growth factor receptor 2 in combination with gemcitabine for patients with advanced pancreatic cancer. *Cancer Science*.

[159] Matejuk A, Leng Q, Chou ST, Mixson AJ (2011). Vaccines targeting the neovasculature of tumors. *Vascular Cell*.

[160] Hicklin DJ, Marincola FM, Ferrone S (1999). HLA class I antigen downregulation in human cancers: T-cell immunotherapy revives an old story. *Molecular Medicine Today*.

[161] Yasuda S, Tsuchiya I, Okada K (2012). Significant clinical response of advanced colon cancer to peptide vaccine therapy: a case report. *The Tokai Journal of Experimental and Clinical Medicine*.

